# Withdrawal of antiepileptic drugs in glioma patients after long-term seizure freedom: design of a prospective observational study

**DOI:** 10.1186/s12883-014-0157-4

**Published:** 2014-08-15

**Authors:** Johan A F Koekkoek, Melissa Kerkhof, Linda Dirven, Jan J Heimans, Tjeerd J Postma, Maaike J Vos, Jacoline E C Bromberg, Martin J van den Bent, Jaap C Reijneveld, Martin J B Taphoorn

**Affiliations:** 1 Department of Neurology, VU University Medical Center, Amsterdam, 1007MB, The Netherlands; 2 Department of Neurology, Medical Center Haaglanden, The Hague, 2501 CK, The Netherlands; 3 Neuro-Oncology Unit, Erasmus MC Cancer Institute, Rotterdam, 3008 AE, The Netherlands

**Keywords:** Brain tumour, Glioma, Epilepsy, Seizures, Antiepileptic drugs, Medication management, Drug withdrawal

## Abstract

**Background:**

Epilepsy is common in patients with a glioma. Antiepileptic drugs (AEDs) are the mainstay of epilepsy treatment, but may cause side effects and may negatively impact neurocognitive functioning and quality of life. Besides antiepileptic drugs, anti-tumour treatment, which currently consists of surgery, radiotherapy and/or chemotherapy, may contribute to seizure control as well. In glioma patients with seizure freedom after anti-tumour therapy the question emerges whether AEDs should be continued, particularly in the case where anti-tumour treatment has been successful. We propose to explore the possibility of AED withdrawal in glioma patients with long-term seizure freedom after anti-tumour therapy and without signs of tumour progression.

**Methods/Design:**

We initiate a prospective, observational study exploring the decision-making process on the withdrawal or continuation of AEDs in low-grade and anaplastic glioma patients with stable disease and prolonged seizure freedom after anti-tumour treatment, and the effects of AED withdrawal or continuation on seizure freedom. We recruit participants through the outpatient clinics of three tertiary referral centers for brain tumour patients in The Netherlands. The patient and the treating physician make a shared decision to either withdraw or continue AED treatment. Over a one-year period, we aim to include 100 glioma patients. We expect approximately half of the participants to be willing to withdraw AEDs. The primary outcome measures are: 1) the outcome of the shared-decision making on AED withdrawal or continuation, and decision related arguments, and 2) seizure freedom at 12 months and 24 months of follow-up. We will also evaluate seizure type and frequency in case of seizure recurrence, as well as neurological symptoms, adverse effects related to AED treatment or withdrawal, other anti-tumour treatments and tumour progression.

**Discussion:**

This study addresses two issues that are currently unexplored. First, it will explore the willingness to withdraw AEDs in glioma patients, and second, it will assess the risk of seizure recurrence in case AEDs are withdrawn in this specific patient population. This study aims to contribute to a more tailored AED treatment, and prevent unnecessary and potentially harmful use of AEDs in glioma patients.

## Background

Epilepsy is common in patients with primary malignant brain tumours, with incidence rates from 20 up to 90%, depending on tumour type, the location of the tumour and its proximity to the cortical gray matter [1]-[3]. The epileptogenicity of a tumour is inversely correlated with its growth rate [4]-[6]. Low-grade gliomas (LGGs), and particularly slow-growing tumours such as gangliogliomas and dysembryoblastic neuroepithelial tumours (DNETs), are the most epileptogenic [7]-[9]. A decrease in seizure frequency is known to contribute to less morbidity and improved quality of life [10],[11]. Therefore, achieving sustained seizure control in these patients is an important issue in brain tumour treatment.

Antiepileptic drugs (AEDs) are the mainstay of epilepsy treatment. However, AEDs may cause side effects and may also negatively impact neurocognitive functioning and quality of life [12]. Moreover, enzyme-inducing AEDs may interfere with chemotherapeutic drugs and corticosteroids and cause additional undesirable adverse effects [1],[13].

It is important to note that anti-tumour treatment, which currently consists of surgery, radiotherapy and/or chemotherapy, may contribute to seizure freedom as well. Retrospective studies on seizure control after surgical resection report seizure freedom after 6–12 months of follow-up in 63-75% of LGG patients [3],[14]-[18]. In a long-term follow-up study of patients with ganglioglioma of which 50% had refractory epilepsy, 85% reported sustained seizure freedom after 5 years [19]. Similar effects on seizure control are observed after radiotherapy or chemotherapy. In the EORTC phase III trial comparing early versus late radiotherapy in LGG patients, after radiotherapy 75% of patients were seizure free, compared to 59% of patients who had not been irradiated [20]. In several smaller series, after temozolomide chemotherapy a more than 50% seizure reduction was seen in 48-59% of LGG patients [21]-[26].

In patients with non-tumour-related epilepsy AEDs are generally discontinued some time after successful epilepsy surgery [18],[27],[28]. AED withdrawal following temporal lobe resection in patients with refractory non-tumoural epilepsy eventually leads to seizure freedom without use of AEDs in 77% of patients [29]. In a review on AED management after epilepsy surgery, AED withdrawal was associated with a lower rate of seizure recurrence compared to AED continuation. Moreover, in 77% of patients whose seizures recurred after AED withdrawal, seizure freedom could be regained after restart of medication [30]. After surgery for paediatric epilepsy, seizure freedom was achieved in 77% of patients who completed AED withdrawal during follow-up. Early AED withdrawal did not affect long-term seizure outcome [31]. In general, epileptologists recommend to start tapering after at least one year of seizure freedom after epilepsy surgery, although the exact timing of AED withdrawal is controversial [32]-[35].

In patients with seizure freedom after anti-tumour therapy the question emerges whether AEDs should be continued, particularly in the case where anti-tumour treatment has been successful. In addition, several studies suggest that the efficacy of AEDs in brain tumour patients is limited. Up to 50% of brain tumour patients still have seizures despite AED treatment [13],[36].

Seizure frequency is likely to increase without AED treatment in glioma patients with ongoing seizures, warranting continued treatment. However, the necessity of AEDs in glioma patients with long-term seizure control is disputable. A few studies support the notion that seizure freedom in brain tumour patients can be maintained without AEDs. In one series 45 out of 62 (73%) children with a brain tumour whose AEDs were withdrawn after anti-tumour treatment became seizure free [37]. Small observational studies on AED use in meningioma and LGG patients showed ongoing seizure freedom after AED withdrawal in a majority of patients [38]. In case seizures reoccur, they are often associated with tumour recurrence [38]. Larger observational studies show that AEDs often are unable to prevent seizure recurrence in patients with renewed tumour growth [16].

Altogether, both studies on seizure freedom after anti-tumour treatment and studies on seizure freedom after epilepsy surgery suggest that an attempt to withdraw AEDs is justified in glioma patients. Therefore, we propose to explore the possibility of AED withdrawal in low-grade and anaplastic glioma patients with long-term seizure freedom after anti-tumour therapy and without signs of tumour progression. As our study population consists of a carefully selected group of glioma patients, we aim to contribute to a more tailored AED treatment, and prevent unnecessary and potentially harmful use of AEDs in glioma patients.

## Methods/Design

### Design and overview

This is a prospective, observational study exploring the decision-making process on the withdrawal or continuation of AEDs in low-grade and anaplastic glioma patients with stable disease and prolonged seizure freedom after anti-tumour treatment, and the effects of AED withdrawal or continuation on seizure freedom. Over a one-year period, we aim to include 100 glioma patients, who will be followed for a duration of at least 24 months. We primarily aim to improve current knowledge on the patient’s and physician’s willingness to withdraw AEDs in brain tumour patients, and to identify the rate of successful AED withdrawals, i.e. that AED’s are withdrawn completely without seizure recurrence. We hypothesize that 1) approximately half of the participants will be willing to withdraw AEDs, and 2) that there will be no significant difference in seizure freedom at last follow-up between patients who have withdrawn and those who have continued their medication.

The study is approved by the institutional review board of the VU University Medical Center (registration number 2013/288).

### Participants

#### Recruitment procedure

We recruit participants through the outpatient clinics of three of the largest tertiary referral centers for brain tumour patients in The Netherlands: one large community hospital (Medical Center Haaglanden, The Hague) and two university hospitals (VU University Medical Center, Amsterdam, and Erasmus MC Cancer Institute, Rotterdam). The three hospitals provide care for approximately one third of the Dutch primary brain tumour population.

One of the co-investigators (JAFK and MK) will explore which patients are potentially eligible for inclusion and will maintain a log of every patient that has been approached to participate. The logs contain the patient’s initials, date of birth, date of screening, and whether the patient has given informed consent, and if not, the reason for non-participation. The co-investigator will inform the treating neuro-oncologists on eligible patients in their outpatients’ clinics. In case the treating neuro-oncologist thinks that AED withdrawal in that particular patient might have serious disadvantages (e.g. due to status epilepticus in medical history, high co-morbidity with recurrent seizures), that patient will not be approached to participate in the study, despite fulfillment of all eligibility criteria. This will be recorded separately.

All eligible patients in whom the physician has no serious objections against AED withdrawal will receive a patient information file. The treating neuro-oncologist will provide additional information and answer questions. Patients who decide to participate will be asked to give informed consent covering four topics: 1) to start the shared decision-making process with their treating physician on the withdrawal or continuation of AEDs and to subsequently implement the joint decision, 2) to collect relevant information from their medical records, 3) to follow-up their seizure status, where possible as a part of the regular out-patient clinic appointments, and 4) to inform other medical specialists or the patient’s general practitioner on the patient’s participation in the study.

#### Eligibility criteria

Patients need to fulfill the following criteria to be included in the study: 1) adult (>18 years); 2) histologically confirmed WHO grade I (pilocytic astrocytoma, pleomorphic xanthoastrocytoma, subependymal giant cell astrocytoma or subependymoma), WHO grade II (astrocytoma, mixed oligo-astrocytoma, oligodendroglioma or ependymoma), or WHO grade III glioma (anaplastic astrocytoma, anaplastic oligodendroglioma, anaplastic oligo-astrocytoma or anaplastic ependymoma); 3) history of epilepsy, defined as the history of at least one seizure except for acute symptomatic seizures, and treatment with AEDs; 4) having undergone anti-tumour treatment (surgical resection, brain irradiation and/or chemotherapy); 5) stable disease with absence of clinical or radiological signs of tumour recurrence, at least during the past 12 months; 6) seizure freedom for at least 12 months from the date of last surgery, irradiation or chemotherapy cycle, *or* seizure freedom for at least 24 months from the last seizure after the last anti-tumour treatment.

### Intervention

The patient and the treating physician make a shared decision on the preferred treatment (continuation or withdrawal of AED). In case of deciding against the withdrawal of AEDs, patient’s and/or physician’s reasons for continuing are explicitly recorded. In case of deciding to withdraw, AEDs are tapered according to a fixed schedule: a step-wise 50% dose reduction takes place every 2 weeks. The minimally required reduction of total daily dose is as follows: valproic acid: 250 mg; levetiracetam: 250 mg; carbamazepine: 100 mg; oxcarbazepine: 150 mg; phenytoin: 50 mg; clobazam: 2.5 mg; lamotrigine: 25 mg; topiramate: 25 mg; clonazepam: 0.25 mg; and lacosamide: 50 mg. When the patient uses 2 or more AEDs, the last add-on AED will be withdrawn first and the primary AED (typically valproic acid, levetiracetam or carbamazepine) will be withdrawn thereafter.

Two groups of patients will be analysed, depending on the outcome of the decision-making process: one group consists of patients whose AEDs will be withdrawn, and the other group consists of patients whose AEDs will be continued. Standard follow-up in both groups takes place after 3, 6, 12, 18 and 24 months. During follow-up, data about AED treatment, including details on the withdrawal where applicable, as well as data about seizure frequency and type, adverse effects, and clinical and radiological brain tumour progression will be collected. The study follow-up will be part of the regular follow-up for those patients at the neuro-oncology outpatients’ clinic. In case of seizure recurrence, AEDs will be adapted or restarted according to the expertise of the treating physician.

### Measurements

#### Baseline characteristics

After the patient has provided informed consent, we will collect information on demographics, seizure onset, seizure type and frequency, date of the last seizure, AED type and dose, adverse effects, as well as data on past anti-tumour treatments, tumour characteristics including histological subtype and location, and neurological and radiological findings.

#### Primary outcome measures

The two primary outcome measures are: 1) the outcome of the shared-decision making on AED withdrawal or continuation, and decision related arguments, and 2) seizure freedom at 12 months and 24 months of follow-up.

#### Secondary outcome measures

Secondary outcome measures are seizure type and frequency in case of seizure recurrence, additional neurological symptoms, adverse effects related to AED treatment or AED withdrawal, other anti-tumour treatments and tumour progression. In case of tumour progression, the time to clinical or radiological progression will be evaluated as well. According to the response assessment in neuro-oncology (RANO) criteria, clinical progression is defined as a definite clinical deterioration that is not attributable to other causes apart from the tumour or a decrease in corticosteroid dose. Radiological progression is defined as a ≥25% increase in the area of the lesions on T2 or FLAIR MRI, or the development of new lesions or increased or new areas of enhancement [23],[39].

At the last follow-up, patient’s decision to withdraw or continue AEDs will be evaluated, using a study-specific questionnaire on patient’s satisfaction with the decision.

### Sample size

Based on the current population of patients with WHO grade I-III glioma with epilepsy receiving treatment in one of the three referral centers, we estimate that in one year a total of 100 patients will be eligible for this study. The number of patients who will actually withdraw their AEDs, will depend on the outcome of the shared decision-making process. Currently, there are no data on patients’ willingness to withdraw AEDs in this specific population. A retrospective study showed that in 42% of patients with intra-axial brain tumours AEDs were withdrawn after surgery as part of routine clinical practice, although patients on prophylactic AED treatment were also included in this analysis [38]. A cautious estimate is that at least 30 percent of the patients will be willing to withdraw AEDs. As this is an observational study that is not primarily aimed at exploring differences between the 2 groups, no formal sample size calculation can be made. A total number of 100 patients, with at least 30 patients in each group, should be sufficient to complete a prediction model, using 3 co-variates. We expect that less than 5% of the eligible patients are unwilling to participate, as this study is mostly part of routine clinical practice and because of the non-interventional character.

### Statistical analyses

#### Descriptive statistics

Baseline patient characteristics and information about seizure freedom and seizure recurrence, AED treatment and withdrawal, tumour symptoms, tumour recurrence and additional anti-tumour treatments will be outlined by means of descriptive statistics.

#### Univariate analysis

With logistic regression analysis, univariate variables (such as age, gender, treatment, seizure type etc.) showing an association (p < 0.10) with the decision to withdraw AEDs will be identified, as well as univariate variables showing an association (p < 0.10) with seizure recurrence.

#### Multivariable analysis

Next, a multivariable logistic regression analysis will be performed to identify independent predictors of the decision to withdraw AEDs. All variables that are univariately associated with this outcome measure will be entered as possible predictors in a multivariable logistic regression analysis. With a backward selection procedure, using a p-value of 0.10 as the removal criterion, significant independent predictors will be identified. Moreover, we will determine predictors of seizure recurrence. A similar analysis will be performed with seizure recurrence as dependent outcome variable. Variables (such as tumour location, pre-operative seizure frequency, response on MRI, etc.) will be entered as possible predictors in both multivariable logistic regression analyses, using the aforementioned criteria. Note that this analysis can only be performed in case of enough statistical power.

#### Interim analysis

The co-investigators (JAFK and MK) will perform an interim analysis when 20 patients who have withdrawn their AEDs have reached at least 12 months of follow-up. The aim of this interim analysis is to evaluate the effect of AED withdrawal on seizure recurrence. In case ≥ 15 patients develop seizure recurrence within 12 months after AED withdrawal, independent of tumour recurrence, we consider the risk of seizure recurrence unacceptably high and the study will be early terminated. The study will also be ended when none of the first 20 included patients is prepared to withdraw AED treatment.

## Discussion

In this report, we describe the design of a prospective, observational study on the withdrawal of AEDs in low-grade and anaplastic glioma patients with prolonged seizure freedom. This study addresses two issues that are currently unexplored. First, it will explore the willingness to withdraw AEDs in glioma patients, and second, it will assess the risk of seizure recurrence in case in this specific patient population AEDs are withdrawn. Furthermore, risk factors for seizure recurrence including tumour progression will be studied.

Given the lack of clear evidence on the feasibility of AED withdrawal after anti-tumour treatment, there are – at this stage – serious ethical objections to the randomisation of patients. Moreover, in clinical practice most patients appear to have strong personal preferences for either withdrawal or continuation of AEDs. As a consequence, we have chosen an observational non-randomised study design. Unlike a randomised controlled study, the observational design allows us to explore the decision-making process with regard to AED treatment. As this study requires minimal additional efforts from the participants, we expect that most patients who are eligible for inclusion will be prepared to provide informed consent. In addition, the limited number of eligibility criteria will result in a high external validity of the study.

The participation of three of the largest referral centers for brain tumour patients contributes to the strength of this study, as this allows us to reach a substantial part of all Dutch low-grade and anaplastic glioma patients. As a consequence of the involvement of patients in the decision-making process, this study is in accordance with current clinical practice.

Based on previous studies on seizure status after anti-tumour treatment or epilepsy surgery, we hypothesize that AEDs can be safely withdrawn in glioma patients who are seizure free and have shown no signs of tumour recurrence. However, we acknowledge that in glioma patients there still is a risk of seizure recurrence, as the epileptogenicity of the tumour may change over time, in particular when the tumour starts growing again [16],[17],[40],[41]. To reduce the risk of seizure recurrence, we will only include patients with clinically and radiologically stable disease and seizure freedom for at least 12 months after the last anti-tumour treatment. In addition, we will include patients who experienced seizures after anti-tumour treatment only after at least 24 months of seizure freedom. To ensure that patients will not be subjected to an unacceptably high risk of seizures, we will perform an interim analysis at the time the first 20 study patients who have withdrawn AEDs will have completed 12 months of follow-up. To prevent excess numbers of patients to be unnecessarily exposed to the intervention, the study will also end if none of the first 20 patients who are included decide to withdraw AEDs.

In summary, we present the design of a prospective observational study aimed at the development of a more targeted and well-considered AED treatment regimen in primary brain tumour patients with epilepsy, and at preventing possibly unnecessary treatment with AEDs. By closely monitoring the decision-making process on AED treatment and a subsequent observation of patient’s seizure status, we will gain more insight into both the willingness of patients and the safety of withdrawing AEDs in glioma patients with a presumably low risk of seizure recurrence. As we expect that AED can be safely withdrawn without substantially increasing the risk of seizure recurrence, this study may lower patients’ and physicians’ threshold to withdraw AEDs in glioma patients. The results of this study may eventually guide future recommendations concerning AED treatment in this specific patient population.

## Abbreviations

AED: Antiepileptic drug

DNET: Dysembryoblastic neuroepithelial tumour

EORTC: European Organisation for Research and Treatment of Cancer

FLAIR: Fluid attenuated inversion recovery

MRI: Magnetic resonance imaging

RANO: Response assessment in neuro-oncology

WHO: World Health Organisation

## Competing interests

The authors declare that they have no competing interests.

## Authors’ contributions

JAFK and MK participated in the design and coordination of the study, and drafted the manuscript. LD, JCR, TJP, JJH and MJBT participated in the study design, and helped to draft the manuscript. MJV, JECB and MJvdB contributed to the study design and critically revised the manuscript. All authors read and approved the final manuscript.
